# Asbestosis in an asbestos composite mill at Mumbai: A prevalence study

**DOI:** 10.1186/1476-069X-4-24

**Published:** 2005-10-31

**Authors:** V Murlidhar, Vijay Kanhere

**Affiliations:** 1 Department of Surgery, LTM Medical College, 1^st ^Floor, College building, Sion, Mumbai 400 022, India; 2 Occupational Health and Safety Centre, Gokuldas Pasta Road, Neelkant Apts, Dadar (E), Mumbai 400 014, India

## Abstract

**Background:**

Of an estimated 100000 workers exposed to asbestos in India, less than 30 have been compensated. The reasons for such a small number are: refusal by management sponsored studies to grant medical certifications to workers suffering from occupational diseases, lack of training for doctors in diagnosis of occupational lung diseases, deliberate misdiagnosis by doctors of asbestosis as either chronic bronchitis or tuberculosis and the inherent class bias of middle class doctors against workers. The aim of the study was to identify workers suffering from Asbestosis (parenchymal and pleural non-malignant disease) among the permanent workers of the Hindustan Composites Factory and assess their disability and medically certify them, whereupon they could avail of their basic rights to obtain compensation and proper treatment.

**Methods:**

The study was conducted by the Occupational Health and Safety Centre and the Workers' Union. Asbestosis was diagnosed if they had an occupational history of asbestos exposure for at least 15 years and showed typical radiographic findings.

**Results:**

Of 232 workers in the factory, 181 participated in the survey. 22% of them had asbestosis. All the asbestos affected workers had at least 20 years of exposure. 7% had rhonchi, 34% had late basal inspiratory rates, 82% had more than 80% of Forced Expiratory Volume in the first second (FEV1)/Forced Vital capacity (FVC) ratio and 66% had FVC less than 80% of the predicted value. On radiology 7% had only pleural disease, 10% had both pleural and parenchymal disease and 82% had only parenchymal disease. The association of pleural disease with chest pain was statistically significant.

**Conclusion:**

We found the prevalence of asbestosis among exposed workers to be less than that anticipated for the number of years of exposure due to "Healthy Worker Effect". We suggest that all affected asbestos workers (including those who have been forced to leave) in India be medically certified and compensated. We also recommend better control of asbestos use in India. We also implore the management to provide all information about the work process and its hazards, conduct medical checkups as mandated by law and give the medical records to the workers.

## Background

There are an estimated 100000 people exposed to asbestos at work in India [1-3]. Many Indian studies have been conducted to estimate the prevalence of Asbestosis in India [4-6]. But less than 30 workers have been compensated even though legislations for compensations in the form of the Workmen's Compensation Act (WC Act) and the Employees State Insurance Act (ESI Act) were enacted in 1923 and 1948 respectively [7]. The reasons for such a small number are: refusal by management sponsored studies to grant medical certifications to workers suffering from occupational diseases, lack of training for doctors in diagnosis of occupational lung diseases, deliberate misdiagnosis by doctors of Asbestosis as either chronic bronchitis or tuberculosis and the inherent class bias of middle class doctors against workers [7-9]. The aim of the study was to identify from among the permanent workers of the Hindustan Composites Factory, those suffering from Asbestosis (parenchymal and pleural non-malignant disease) and assess their disability so that they could be medically certified and avail of their basic rights to obtain compensation and proper treatment.

The case study, Hindustan Composites, then called Asbestos Magnesia and Friction Material (AM and FM) was established in Sewri, Mumbai in the year 1949. In 1956, with a change of name to Hindustan Ferodo, it was shifted to its present site in Ghatkopar, Mumbai. In 1990, the ownership changed and the company is now called Hindustan Composites. In 2003, the management declared a lockout that lasted for seven and a half months. The workers' union struggled during this time and after an agreement to reduce the workforce, production began in June 2004. The company started production again with 232 permanent workers and around 100 contract (temporary) workers. 110 workers were coerced to resign under the so-called Voluntary Retirement Scheme (VRS).

The workers union of the Hindustan Composites Asbestos Factory, the Krantikari Kamgar Union, approached the Occupational Health and Safety Centre (OHSC), Mumbai in March 2004 to study the prevalence of Asbestosis amongst its workers and get medical certification of affected workers.

The Occupational Health and Safety Centre (OHSC) [10], established in 1988, is a voluntary organization working with workers and unions. The OHSC has in the past conducted studies on occupational diseases and has helped more than thousand workers to claim compensation for Byssinosis, Noise-induced hearing loss, Occupational asthma, Acid burns, Radiation injuries and other occupational injuries [10-14].

### Work Process

The following section describes the factory processes that lead to asbestos production. The management refused permission to conduct the survey inside the factory premises and did not cooperate in the survey conducted outside the factory's gates. They had not given the workers any information regarding the health effects of asbestos and other hazardous materials used in the production process. The workers were not involved in any of the decision-making processes involved in procuring the raw material and in the work process. Hence, the following information regarding the production process is from the knowledge that the workers have gathered during their years of work.

There are four main departments in the factory: Asbestos Textiles, Compressed Asbestos Fiber (CAF), Brake and Clutch Lining (BCL) and Goods Receiving Section (GRS). There are other allied departments such as Maintenance, Security etc. Asbestos Textiles, Compressed Asbestos Fiber (CAF), Brake and Clutch Lining (BCL) are the "dusty sections" where the exposure to asbestos dust is the highest.

White asbestos is brought to the factory in bags. It is in the form of minute fibers that appears like white powder or lumps. It enters the process in the factory at two stage points.

At one of the stage points, Asbestos is mixed with a viscose material and then sent to the Carding Section. In the Carding Section, slivers or wicks are produced. A sliver is a loose, thin continuous fiber ready to be drawn and twisted. A wick is a piece of cord or tape. These are transferred to the Frame Section. Here, yarn is produced by plying/winding of 2, 3 or 4 fibers together. From the Frame Section the material is sent to the Plating Section to produce rope, to the Weaving Section to make cloth or rolls, and to the BCL department. In the BCL department, the above material is mixed with varnish, heated and cooled. Then, surface grinding takes place.

At the other stage point, Asbestos fiber is mixed with resins or rubber and is processed in a mixer with spikes. The rotating mixer is heated. The soft material produced is sent between two rolls/calendars. One roll is heated and the other is chilled. Asbestos sheets are produced when this material comes out of the rolls.

## Methods

Worker-activists and one of the authors, V. Murlidhar (hereinafter VM), discussed the modalities and necessary steps in diagnosis of Asbestosis. The study was done from 10^th ^of November 2004 to 13^th ^of November 2004 at the gates of the factory. Workers were informed and came as per their convenience for the check up. The response from the workers was very good and out of the 232 permanent workers, 181 attended the camp. The contract workers have an insecure tenure and were apprehensive. As a result, they did not present for the examination.

One of the authors (VM) was present when the questionnaire was filled for every worker. The questionnaire prepared by us was similar to that used in previous published studies on surveys of occupational lung diseases [12]. A detailed occupational history of exposure to asbestos was taken as per standard guidelines [15]. Symptoms of dyspnoea, chest pain and chest tightness were recorded. We physically examined workers for clubbing, end-inspiratory basal crackles and rhonchi, which have prognostic significance [16,17]. Smoking score was defined as number of cigarettes smoked per day times the number of smoking years.

Lung function tests (PFTs) were performed, as in previous studies of occupational lung disease by the OHSC [12]. The lung function tests were carried out using a Wright ventilometer (VM1) which gives digital readings for Forced Vital capacity (FVC), Forced Expired volume in the first second (FEV1), their ratio and Peak flow rate (PEF).

Three readings of PFT were taken and the consistent value chosen. Prediction equations for Indian subjects were used to calculate the expected Forced Expiratory Vital capacity in the First Second (FEV1) and Forced Vital capacity (FVC values) [18]. The equations used were as follows:

Females

FEV1: 0.0274 H – 0.0103 A – 1.995

FVC: 0.0370 H – 0.0070 – 3.197

Males

FEV1: 0.0396 H – 0.0212 A – 3.130

FVC: 0.0603 H – 0.0136 – 4.488

H= Height in cms

A= Age in years

Chest radiographs were taken as per prescribed specifications and classified with due regard to quality as per the International Classification of Radiographs of Pneumoconiosis (ILO classification), [19,20] also see additional file 1: Chest radiograph classification. The radiographs were studied and classified by one of the authors (VM). The ILO classification profusion score of 1/1 and greater was considered positive for diagnosis of Asbestosis, which is the 1986 guideline laid down by the American Thoracic Society [19].

High Resolution Computerized Tomography (HRCT) was done in only one patient who was suspected to have lung cancer. The other suspected patient of pleural cancer opted not to be investigated further.

The data was fed into *EPI6 *for MSDOS statistical software. The statistical test used when appropriate was the Chi Square test.

### Diagnostic Criteria for Asbestosis

Asbestosis was diagnosed if they had an occupational history of asbestos exposure for at least 15 years and showed typical radiographic findings.

Clinical examination findings were used only as prognostic indicator for treatment. Pulmonary function testing was used for impairment assessment as per standard criteria, [13,21] also see additional file 2: Pulmonary Impairment assessment guidelines.

## Results

### Asbestosis Distribution

The workers of Textile, BCL and CAF departments (which have higher dust exposure) constituted 81% of the workers examined, while workers in Maintenance and other ancillary department (which have lesser dust exposure) accounted for the remaining 19% (Table I).

**Table 1:** Distribution of workers among departments: n = 181

Department	**Dusty Sections**			Other Sections		**Total**
		
	Textile	BCL	CAF	Maintenance	Other	
Number of Workers	93	18	37	11	22	181
Number of Workers affected by Asbestosis	21(23%)^a^	7(39%)^a^	6(16%)^a^	3(27%)^a^	4(18%)^a^	41(23%)^a^

^a ^Number of workers affected by Asbestosis (% age of total number of workers in the department)

There were 41(23%) Asbestosis cases among the workers. Of them 34(82%) were from the dusty sections (Table I). Only 7(18%) were from the other departments but it was not statistically significant.

All workers had at least 20 years of exposure, 51% had an exposure of more than 30 years. All workers were above 40 years of age, 52% of the workers were older than 50 years of age. The mean age was 54 years.

### Symptoms and Signs

Only 4% of the total workers reported history suggestive of chronic bronchitis. Of the total workers, 77% were non-smokers, 13% had a smoking score of less than 100 and only 8% had a smoking score of more than 100. 20 percent of the Asbestosis-affected workers gave history of smoking. Among the non-smokers, 33% (11) had FEV1 less than 80% of the predicted value.

Half of the workers reported mild dyspnoea grade 1 and only 3% reported grade 2 dyspnoea. Of these 55% had FVC less than 80% of the predicted value.

Mild chest pain or tight chest was reported by 50% of the workers. The chest discomfort was not continuous in anyone. The chest pain symptoms were reported for less than 5 years in 39%, and only 11% had the symptom for more than 5 years. Only 2% of the total workers had pulmonary tuberculosis, which was treated fully.

On clinical examination of the Asbestosis cases, none had clubbing, only 7% had rhonchi which were occasional and 34% had late inspiratory basal crackles. Two of the Asbestosis affected workers had pleural/lung tumors in addition to having Parenchymal Asbestosis. One of them declined further investigation, the other case was investigated by HRCT and lung biopsy, and lung cancer (T3, N2, and M0) was proved. Another worker died of cardio pulmonary failure within weeks of the completion of the study and certification as Asbestosis. The average disability percent of the affected workers was 32%.

### Lung Function Tests (FEV1, FVC, and FEV1/FVC)

Of the 181 workers tested, 62% had abnormal FVC (less than 80% of predicted), 15% had abnormal FEV1 (less than 80% of predicted), 79% had more than 80% of FEV1/FVC.

Of the 41 Asbestosis affected workers, 66% had FVC less than 80% of predicted, 37% had FEV1 less than 80% of predicted and 82% had more than 80% of FEV1/FVC. (Table II)

**Table 2:** Lung Function in the Asbestosis cases. n = 41

**Pulmonary Function**	**Percentage Predicted FVC**		**Percentage Predicted FEV1**		**FEV1/FVC %**		
	
	< 80%	> 80%	<80 %	> 80%	< 80%	80–90%	> 90%
Number of workers	27(5)^a^	14(2)^a^	15(3)^a^	26(4)^a^	7(2)^a^	19(2)^a^	15(3)^a^
Percentage	66%	34%	37%	63%	18%	46%	36%

^a ^Number of workers checked (Number of Smokers)

### Chest X-ray

Pleural disease was identified in 17% of the affected workers, parenchymal disease in 92% of the affected workers (82% had only parenchymal disease) and 7% had only pleural disease. (Table III)

**Table 3:** Radiological findings of Asbestosis cases: n = 41

**Pleural Disease**			**Parenchymal disease**			
All Pleural	Only Pleural	Pleural and Parenchymal	All Parenchymal	s/s^a^	s/t^a^	t/t^a^
7(17%)^b^	3(7%)^b^	4(10%)^b^	38 (92%)^b^	27(65%)^b^	8(19%)^b^	3(7%)^b^

^a- ^s/s, s/t, t/t are classification of opacities as per ILO criteria [20] ^b^**- Number of cases (%age)**

Of the 7 cases with pleural disease 5 reported chest pain where as only 1(one) with parenchymal disease reported chest pain. This is statistically significant (p = 0.05).

## Discussion

It is estimated that 6000 workers are directly exposed and nearly 100000 workers indirectly exposed to asbestos [1]. The prevalence rate of Asbestosis in our study was 23%, which is less than the expected prevalence among workers exposed to asbestos for more than 20 years [16,22]. Many studies reported a prevalence of above 70% among workers exposed to asbestos for more than 20 years [16,19,22].

The reasons for the lower prevalence found in our study are many. The primary reason is the "healthy worker effect". Many affected workers have been forced to leave the company or to take voluntary retirement (VRS). Some may even have died due to the disease. Hence, the workers remaining in the factory are relatively healthy workers. As in most industries, workers who are casual or temporary do the hazardous jobs. However, since their livelihood is at stake, they would not come for the survey. Hence, exclusion of the casual workers in whom the prevalence rate would probably be higher is another reason for the lower prevalence in our study. A chest film clearly showing the characteristic signs of Asbestosis in the presence of a compatible history of exposure is adequate for the diagnosis of the disease. Further imaging procedures like an HRCT are not required [19]. Nevertheless, a high resolution CT (HRCT) might have picked up more cases of parenchymal Asbestosis [23,24]. Financial constraints limited the number of workers who could undergo HRCT. It was also beyond our capacity to identify workers who had left their jobs or had retired. These could have given us a truer prevalence rate.

Nearly half of the workers reported dyspnoea and 55% of them had FVC less than 80% of the predicted value. 11–17% reduction in the FVC has been reported in asbestos workers who report dyspnoea [25]. Even report of mild dyspnoea is important, as has been indicated in another study where 33% of asbestos workers reporting mild dyspnoea had diminished FVC [26]. In our study 34% had basal rates, which increases the risk of asbestos-related mortality [16,19].

In our study parenchymal changes are more common that pleural changes. This is similar to another Indian study on asbestos prevalence where they found that parenchymal changes are six times more common than pleural changes in Asbestosis [4]. In our study, there was a statistically significant association of chest pain with pleural changes on radiographs. This is consistent with another study wherein nearly half of the workers with pleural disease reported chest pain [26]. Rapidly progressive or severe chest pain should raise clinical suspicion of malignancy [19].

Physical findings in Asbestosis include basilar rates, often characterized by end-inspiratory crackles (rates). In some cases of advanced Asbestosis, finger clubbing may be present [16,19]. Physical findings of crackles, clubbing, or cyanosis are associated with increased risk for asbestos-related mortality [16,17]. In our study nearly 40% of Asbestosis workers had either rates or rhonchi. However, physical findings have low sensitivity and hence limited clinical utility [19].

Tuberculosis (TB) was noted in 2% of cases. It is important to clearly diagnose TB, since Asbestosis in India has been misreported as TB in the past [7]. We also found two cases of pulmonary/pleural tumors. This is important because not a single case of occupational lung cancer has been compensated in India, to date.

The lung function impairments in Asbestosis affected workers in our study were typical: majority of the workers 66% had a restrictive pattern with diminished FVC, and 37% had an obstructive pattern of decreased FEV1. As with other interstitial lung diseases, the classic finding of Asbestosis is a restrictive impairment as in our study. Mixed restrictive and obstructive impairment is also frequently seen; in contrast, isolated obstructive impairment is unusual [19]. Most workers affected by Asbestosis (82%) also had FEV1/FVC ratio above 80%. This increase in ratio has been noted in the past [27]. Demonstration of functional impairment is not required for the diagnosis of a nonmalignant asbestos-related disease, but where present should be documented as part of the complete evaluation [19]. It contributes to the diagnosis in defining the activity of the diseases and the resulting impairment can be quantified for compensation purposes [19,21].

It is reported that nearly 50% of Asbestosis workers have a FVC less than 80% of the predicted value. Hence, we tabulated the ventilatory impairments using this criteria [19]. Another reason for using the 80% of expected FVC and FEV1 criteria for tabulation lay in the use of this cut-off limit by the assessments of impairments due to pulmonary disease [21].

It is never enough to emphasize that institutional deficiencies within the medical system and the industry management are the primary contributors to occupational diseases like Asbestosis. Disability assessment is an important responsibility of the physician, yet, it is not routinely taught in medical schools [11]. Despite compensation being a legal right in India, the affected worker cannot hope to gain compensation without certifications of the resultant impairment as an occupational disease [11]. As per Indian Law, it is mandatory for the management to give detailed information about the work processes and the effects of the hazardous processes on one's health to workers in their local language. This was not done by the management. Such an act is indubitably unethical [8]. Forcing the management to pay compensation to workers will induce the owners to employ the safety measures and precautions that are mandated by Indian Law.

## Conclusion

We found the prevalence of Asbestosis among exposed workers to be less than that expected for years of exposure. This is mainly due to the "healthy worker effect" (i.e., most of the affected workers in past years have either died or have been removed from employment due to lockouts). Some have opted for early retirement coerced by the management.

There are less than 30 cases of Asbestosis compensated in India among the 100000 exposed workers. Many must have died of the disease or of lung or pleura cancer. Workers concerned with asbestos are to be medically checked by the management every year while continuing in such a job *and *after he has ceased to work in such a job. This is a specific responsibility of the occupier of any factory having any hazardous process. All the workers who have left a job involving asbestos, under VRS or otherwise, need to be medically checked, once a year at the very least. This is their legal right.

The diagnosis of Asbestosis, in particular, imposes a duty on the doctor to inform the patient that he or she has a disease that is work-related. The duty extends to reporting the disease and to inform the patient that he or she may have legal or adjudication options for compensation. The role of the physician in this compensation process includes performing an objective evaluation of impairment consistent with the rules of the specific compensation system [10,11,13-15].

We recommend better control of asbestos use in India. We also recommend that the management give all information regarding the hazardous processes and their medical records after conducting the mandatory annual medical checkups as is mandated by law.

## List of abbreviations

AM and FM: Asbestos Magnesia and Friction Material

BCL: Brake and Clutch Lining

CAF: Compressed Asbestos Fiber

GRS: Goods Receiving Section

CAF: Compressed Asbestos Fiber

ESI Act: Employees State Insurance Act

FVC: Forced Vital capacity

FEV1: Forced Expiratory Volume in the first second

HRCT: High Resolution Computerized Tomography

ILO classification: International Classification of Radiographs of Pneumoconiosis

OHSC: Occupational Health and Safety Centre

PFTs: Lung function tests

PEF: Peak flow rate

TB: Tuberculosis

VRS: Voluntary Retirement Scheme

WC Act: Workmen's Compensation Act

## Competing interests

The author(s) declare that they have no competing interests.

## Authors' contributions

VM conceived the study, supervised all aspects of its implementation, and led the writing of the manuscript. VK assisted with the study and contributed to the design and analysis of all study components. All authors helped to interpret findings and review drafts of the manuscript.

## Supplementary Material

Additional File 1 Chest radiograph classification format as per ILO classification. Description of Data: This table is the specified format for recording chest radiographs as per the ILO guidelines for classification of pneumoconiosis. Click here for file

Additional File 2 Impairment Assessment guidelines used for calculating disability of affected workers. Description of Data: These are the guidelines used for assessment of respiratory impairment. Click here for file
