# Supplementary material for: Asbestosis in an asbestos composite mill at Mumbai: A prevalence study
Source: Environ Health. 2005 Oct 31;4:24. doi: 10.1186/1476-069X-4-24 (PMC1289287; doi:10.1186/1476-069X-4-24)
Supplement: Additional File 1 — Chest radiograph classification format as per ILO classification. Description of Data: This table is the specified format for recording chest radiographs as per the ILO guidelines for classification of pneumoconiosis. [file 1476-069X-4-24-S1.pdf]

Chest radiograph classification format as per ILO classification

Film Quality	Grade 1-4<br>Comment made (y/n)		
Small Opacities	Profusion | R | | L<br>Shape and size		
Large opacities	Presence (y/n)<br>Size (A,B,C)		
Pleural thickening Chest wall Circumscribed Presence (y/n)	R | L<br>Face on (y/n)<br>Width (a,b,c)<br>Extent (1,2,3)		
Pleural thickening Chest wall Diffuse Presence (y/n)	R | L<br>Face on (y/n)<br>Width (a,b,c)<br>Extent (1,2,3)		
Pleural thickening (Diaphragm)	Presence (y/n)<br>R | L		
Costophrenic Angle Obliteration	Presence (y/n)<br>R | L		
Pleural Calcification	Presence (y/n)		
Chest wall<br>Diaphragm<br>Other<br>Extent (1,2,3)	R | L		
Symbols	Used (y/n)		
Comments	Made (y/n)		
