# Supplementary material for: Asbestosis in an asbestos composite mill at Mumbai: A prevalence study
Source: Environ Health. 2005 Oct 31;4:24. doi: 10.1186/1476-069X-4-24 (PMC1289287; doi:10.1186/1476-069X-4-24)
Supplement: Additional File 2 — Impairment Assessment guidelines used for calculating disability of affected workers. Description of Data: These are the guidelines used for assessment of respiratory impairment. [file 1476-069X-4-24-S2.pdf]

Impairment Assessment guidelines used for calculating pulmonary disability of affected workers.

	Class I Upto 25% Impairment	Class II 26% - 50%	Class III 51% - 75%	Class IV 76% - 100%
Dyspnoea	When it occurs, is consistent with the circumstances of activity	Does not occur at rest and seldom occurs during the performance of the usual activities of daily living. The patient can keep pace with persons of same age and body built on the level without breathlessness but not on hills or stairs	Does not occur at rest but does occur during the usual activities of daily living. However, the patient can walk a mile at his own pace without dyspnoea although he cannot keep pace on the level with others of the same age and body build	Occurs during such activities as climbing one flight of stairs or walking 100 yards on the level, on less exertion, o even at rest
FEV_1	> 80% of predicted	60- 79% of predicted	51 – 59% of predicted	< 50% of predicted
	AND	OR	OR	OR
FVC	> 80% of predicted	60 – 79% of predicted	51 – 59% of predicted	< 50% of predicted
	AND	OR	OR	OR
$(FEV_1/FVC) \times 100$	> 75% of predicted	60 – 74% of predicted	41 – 59% of predicted	< 40% of predicted
